# Draft Genome Sequences of Three Human Pathogenic Acinetobacter baumannii Strains

**DOI:** 10.1128/MRA.01742-18

**Published:** 2019-04-04

**Authors:** Masoumeh Madhi, Troels Ronco, Alka Hasani, Rikke H. Olsen

**Affiliations:** a Immunology Research Center, Tabriz University of Medical Sciences, Tabriz, Iran; b Department of Medicinal Microbiology, Tabriz University of Medical Sciences, Tabriz, Iran; c Student Research Committee, Tabriz University of Medical Sciences, Tabriz, Iran; d Department of Veterinary and Animal Sciences, Faculty of Health and Medical Sciences, University of Copenhagen, Copenhagen, Denmark; University of Maryland School of Medicine

## Abstract

Acinetobacter baumannii is an opportunistic human pathogen with the ability to develop multiple resistances against the main antibiotic classes. It causes nosocomial infections, especially in intensive care units. Here, we present the draft genome sequences of three isolates (AB1, AB2, and AB3) from humans, collected from two hospitals in Tabriz, Iran.

## Abstract (precis)

Acinetobacter baumannii is an opportunistic human pathogen with the ability to develop multiple resistances against the main antibiotic classes. It causes nosocomial infections, especially in intensive care units.

## ANNOUNCEMENT

Acinetobacter baumannii is a Gram-negative coccobacillus. It causes nosocomial infections, such as bacteremia and soft tissue and skin infections, especially in burn patients (1). Because of the extraordinary ability of *Acinetobacter* to develop resistances against multiple antibiotic classes, clinicians face a major problem, especially in intensive care units (ICUs). Here, we present the draft genomes of three A. baumannii isolates (AB1, AB2, and AB3) from two hospitals in Tabriz, Iran.

Strain AB1 was isolated from the blood of a newborn child in the neonatal ICU at the children’s hospital in Tabriz, Iran, using BD BACTEC blood culture medium. Strain AB2 was collected from the tracheal tube of a male using a sterile tube with physiological serum (0.9% NaCl), in the ICU of Sina Hospital in Tabriz. Strain AB3 was isolated from a scar sample of a 4-year-old child in the burn ICU of Sina Hospital, using Amies transport medium. The samples followed the routine methodology of diagnostic procedures and grown overnight at 37°C on blood agar and MacConkey agar (MA). Subsequently, nonlactose A. baumannii colonies were identified with conventional biochemical tests. Single colonies on Mueller-Hinton agar (Sigma-Aldrich, St. Louis, MO, USA) were then selected, and DNA extraction was performed with the Maxwell system (Promega, Madison, WI, USA) including the RSC cultured cell kit according to the manufacturer’s instructions. Colonies were identified to the species level with PCR of the *rpoB* gene (1). Genomic DNA was extracted as previously described, and the Nextera XT kit (Illumina, San Diego, CA, USA) was used for preparing DNA libraries according to the manufacturer’s instructions. The libraries were paired-end sequenced (2 × 251-bp read length) with Illumina’s MiSeq platform. The quality of the FastQ files was assessed with FastQC version 0.11.8 (http://www.bioinformatics.babraham.ac.uk/projects/fastqc/), and all per-base-sequence quality score means were greater than 20. The files were *de novo* assembled in the CLC bio Genomics Workbench version 7.04 (Qiagen, Aarhus, Denmark) with a minimum contig length of 500 bp. Assembly metrics can be found in Table 1. The contigs were annotated with the NCBI Prokaryotic Genome Annotation Pipeline (2), and the number of protein coding DNA sequences (CDSs) are shown in Table 1. The sequence types (STs) were determined at pubMLST (3). Strains AB1 and AB2 belonged to ST2, whereas strain AB3 belonged to ST136. Previously, these STs have been reported from China and Kuwait (4–6). In addition, antibiotic resistance genes were identified with ResFinder version 3.1 (7), and various types of resistance genes were found. Aminoglycoside, beta-lactam, and tetracycline resistance genes were identified in all three strains, whereas only strains AB1 and AB2 carried macrolide resistance genes. A single sulfonamide resistance gene was found in strain AB3. All genes were identified with a nucleotide identity of 90% and query sequence coverage of 90%.

**TABLE 1:** Assembly and annotation metrics for strains AB1, AB2, and AB3

Strain	Avg coverage (×)	No. of contigs	*N*_50_ (kb)	Assembly length (kb)	G+C content (%)	No. of protein CDSs
AB1	70	365	24.2	3,880	39.0	3,709
AB2	76	289	31.9	3,864	39.0	3,666
AB3	89	243	36.3	4,121	38.7	3,955

### Data availability.

The draft genome sequences of strains AB1, AB2, and AB3 have been deposited in DDBJ/ENA/GenBank under the accession numbers RXLS00000000, RXLT00000000, and RXLU00000000, respectively. The versions described here are the first versions, RXLS01000000, RXLT01000000, and RXLU01000000, respectively. The Sequence Read Archive accession numbers are SRR8380009, SRR8380008, and SRR8380007, respectively.
